# Discovering novel driver mutations from pan-cancer analysis of mutational and gene expression profiles

**DOI:** 10.1371/journal.pone.0242780

**Published:** 2020-11-24

**Authors:** Houriiyah Tegally, Kevin H. Kensler, Zahra Mungloo-Dilmohamud, Anisah W. Ghoorah, Timothy R. Rebbeck, Shakuntala Baichoo

**Affiliations:** 1 Department of Digital Technologies, FoICDT, University of Mauritius, Réduit, Mauritius; 2 Dana Farber Cancer Institute, Harvard TH Chan School of Public Health, Boston, MA, United States of America; King's College London, UNITED KINGDOM

## Abstract

As the genomic profile across cancers varies from person to person, patient prognosis and treatment may differ based on the mutational signature of each tumour. Thus, it is critical to understand genomic drivers of cancer and identify potential mutational commonalities across tumors originating at diverse anatomical sites. Large-scale cancer genomics initiatives, such as TCGA, ICGC and GENIE have enabled the analysis of thousands of tumour genomes. Our goal was to identify new cancer-causing mutations that may be common across tumour sites using mutational and gene expression profiles. Genomic and transcriptomic data from breast, ovarian, and prostate cancers were aggregated and analysed using differential gene expression methods to identify the effect of specific mutations on the expression of multiple genes. Mutated genes associated with the most differentially expressed genes were considered to be novel candidates for driver mutations, and were validated through literature mining, pathway analysis and clinical data investigation. Our driver selection method successfully identified 116 probable novel cancer-causing genes, with 4 discovered in patients having no alterations in any known driver genes: MXRA5, *OBSCN*, *RYR1*, and *TG*. The candidate genes previously not officially classified as cancer-causing showed enrichment in cancer pathways and in cancer diseases. They also matched expectations pertaining to properties of cancer genes, for instance, showing larger gene and protein lengths, and having mutation patterns suggesting oncogenic or tumor suppressor properties. Our approach allows for the identification of novel putative driver genes that are common across cancer sites using an unbiased approach without any *a priori* knowledge on pathways or gene interactions and is therefore an agnostic approach to the identification of putative common driver genes acting at multiple cancer sites.

## Introduction

Cancer arises from genomic alterations that give cells a selective advantage for abnormal growth. These somatic alterations include single-nucleotide variants (SNVs), insertions, deletions and copy-number variants (CNVs), which accumulate in the genome over time [1]. Exploration of the cancer genome have revealed important insights into cancer driver mutations that are responsible for oncogenesis, tumor invasion, and metastatic potential [2, 3]. Targeted therapies have emerged from the development of drugs acting specifically against driver mutations. For example, basket trials have been undertaken that target therapeutic interventions at driver mutations rather than a specific anatomic tumor site [4]. Given the extreme intra- and inter-individual genomic heterogeneity of most cancers, the limited knowledge of cancer driver genes limits the development and application of targeted therapies.

Advances in high-throughput sequencing technologies led to the establishment of international cancer genomics data initiatives including the International Cancer Genomics Consortium (ICGC), The Cancer Genome Atlas (TCGA) and American Association for Cancer Research (AACR) Project GENIE [5–7]. These databases provide the opportunity to identify targetable alterations [8, 9] and improve our understanding of the genetic basis of cancer development, progression, and therapy [7, 10–13]. Despite this progress, it is likely that additional genomic drivers of cancer exist. For example, exome sequences from more than a thousand prostate cancer samples have recently revealed new oncogenic drivers [14] that suggested a large number of mutations, occurring at lower frequencies than previously thought, could potentially be therapeutically targeted for improved clinical outcomes. It is likely that this phenomenon also exists for other cancer types.

A challenge for genomic analysis is to distinguish driver mutations from the complex heterogeneous background landscape of “passenger” somatic alterations, which are not causative of oncogenesis [15]. Various tools and strategies have been developed to identify driver mutations from passenger alterations [16]. The aim of the present research is to agnostically identify new candidate driver mutations by considering genomic commonalities between a number of cancer types. While the co-analysis of genomic and transcriptomic information for identification of cancer drivers has helped to elucidate driver mutations and pathways in individual cancers [17–19], our research aims to identify common events occurring across a number of tumour types. The concurrent study of different cancers together can reveal driver mutations that are not detected in a single cancer site. Pan-cancer studies have mostly used mutation frequency-based approaches to detect driver mutations [20]. However, use of mutation frequency alone may result in erroneous inferences about driver mutation status [21]. By integrating the intersectional analysis of mutation and gene expression profiles to a pan cancer approach, it may be possible to uncover candidate driver mutations that might have been hidden within the “long tail” of oncogenic drivers [14].

We hypothesize that genomic alterations causing the significant over- or under-expression of genes are more likely to represent cancer drivers. For example, data considering mutations that affect gene expression levels have been used to identify cancer drivers previously in glioblastoma [17]. As a proof of concept, we applied this strategy on three types of cancer: breast, ovarian and prostate. These cancers have high incidence worldwide, are considered to be hormone-related cancers, and have common low-penetrance susceptibility variants [22]. Hence, this shared etiology raises the possibility that all three cancers are under the influence of common oncogenetic pathways.

## Results

### Selection of candidate cancer driver genes

**Fig 1:**
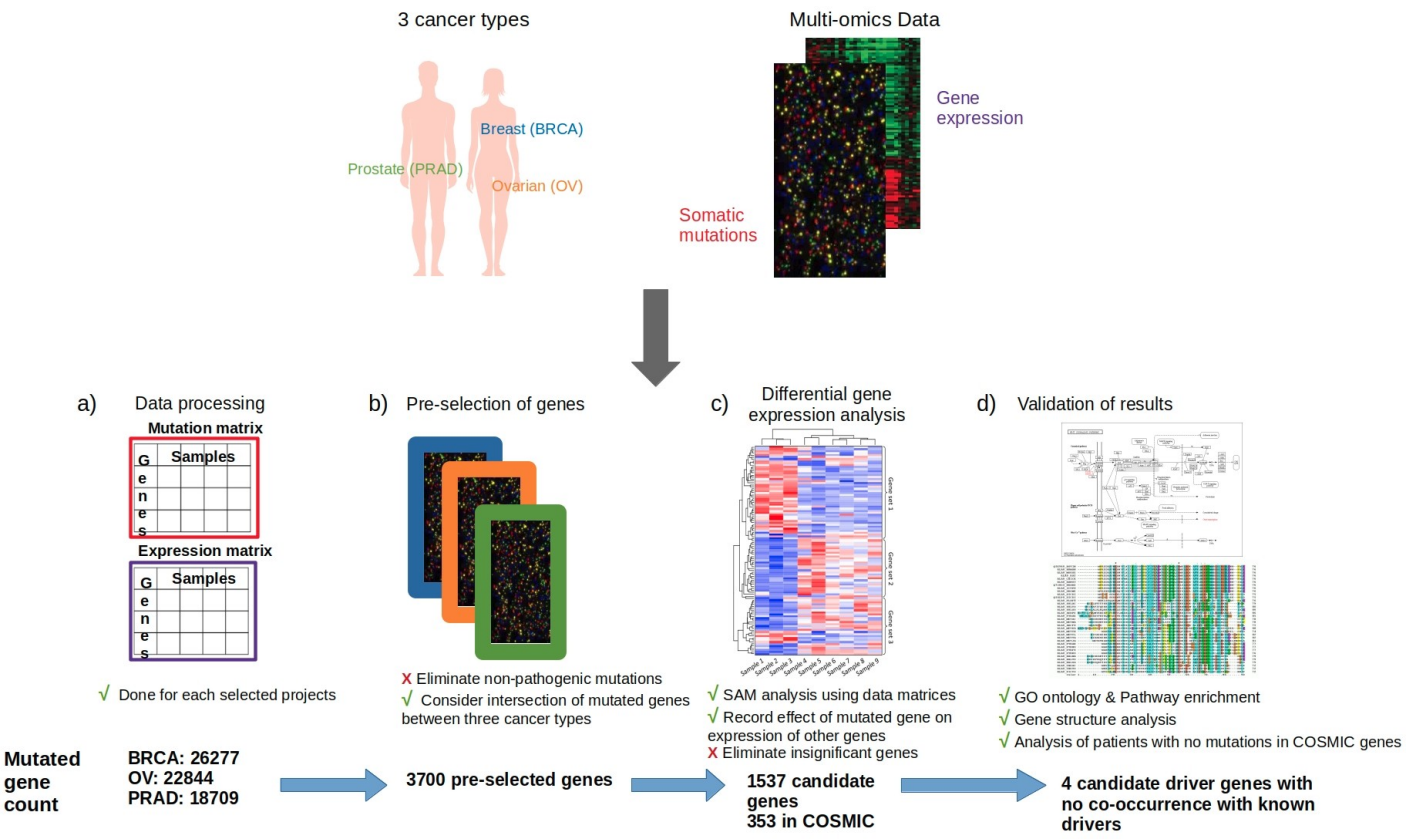
Summary of approach. In this research, we have identified novel driver mutations by computing the intersection of mutational and gene expression data, and later validated candidate driver mutations using literature mining and pathway analysis. This study pooled together mutational and gene expression data from three cancer types (breast, ovarian and prostate cancers) from TCGA datasets to demonstrate an unbiased approach for cancer-driver gene selection. a) Mutation and gene expression data are processed into mutation and expression matrices for integrative data analysis; b) Pre-selection of genes includes the exclusion of non-pathogenic variants, and an intersection of the remaining mutated genes in the three cancer types (TCGA datasets). c) The pre-selected genes are investigated for their effect on gene expression (as a measure of functionality) by performing differential gene expression analysis. d) The final genes are subjected to gene ontology and pathway enrichment for validation, and the same analysis is performed on patients.

**Fig 2:**
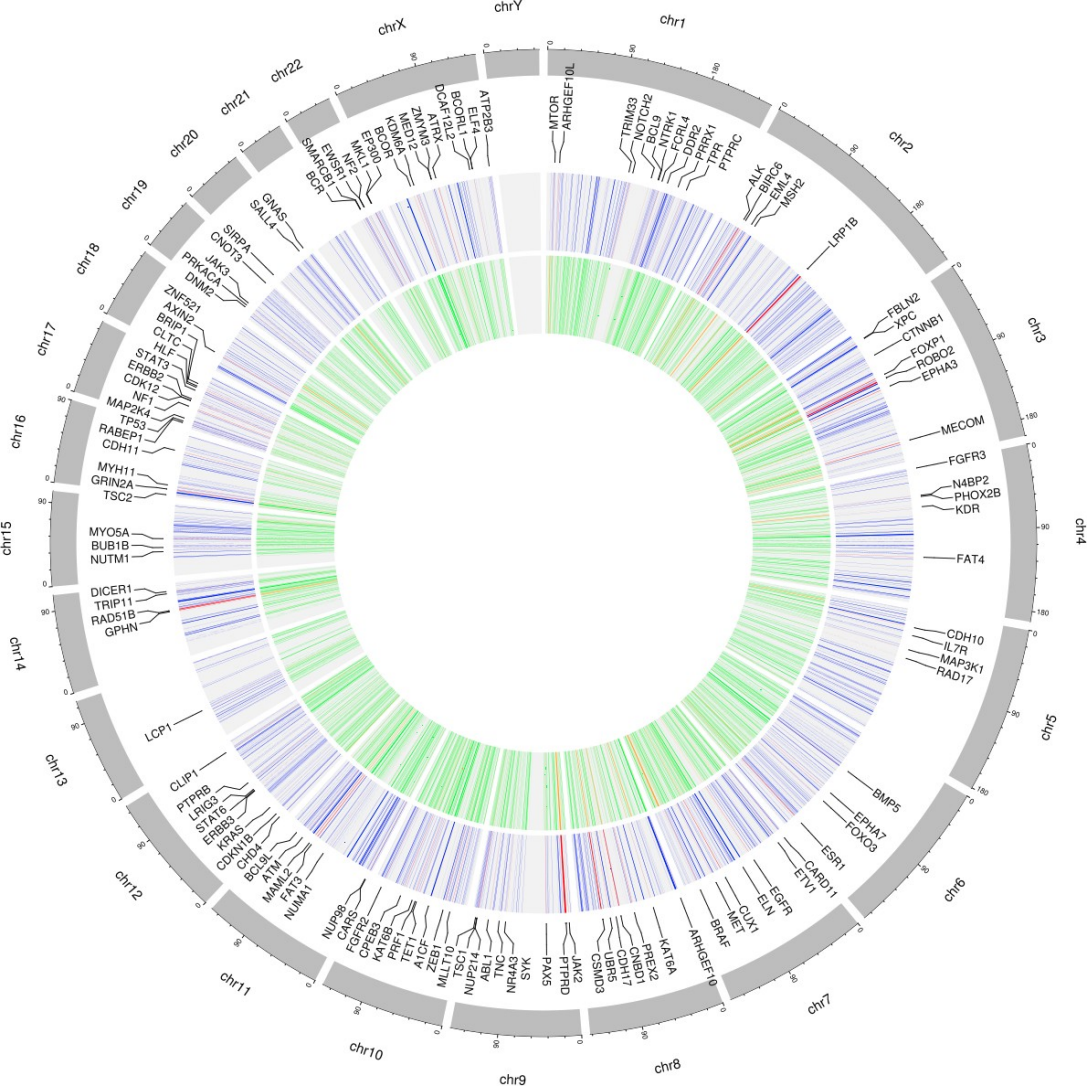
Mutated genes of interest. Circos plots showing the distribution, across the human genomes, of the 3700 pre-selected genes (inner circle) commonly mutated BRCA-US, OV-US, and PRAD-US cancer data sets, including COSMIC (orange) and non-COSMIC (green) genes (red); The second circle from the middle shows the 1537 cancer-causing candidate genes, with non-COSMIC genes in blue, and COSMIC genes in red labeled with their gene names.

Breast, ovarian, and prostate cancers were selected for this study as a proof of concept because an initial exploration of their mutational profiles revealed that they share about 50% of their top mutated genes in tumor tissue (S1 Fig). Somatic mutation data from TCGA consisted of 26,277 mutated genes for breast cancer (BRCA-US), 22,844 for ovarian cancer (OV-US) and 18,709 for prostate cancer (PRAD-US) (Figs 1 and 2A). Initial selection of genes (genes mutated in all three cancer types, and exclusion of non-pathogenic variants) yielded a list of 3700 pre-selected mutated genes (Fig 1B).

Upon differential gene expression analysis of tumor samples harboring alterations in these genes, a range of effects on the gene expression of other genes in each data set was observed (Fig 2). From these results, a gene was defined as a candidate cancer-causing driver gene if it affected the expression of other genes in all three cancer types when mutated. Based on these criteria, 1537 genes were selected as candidate cancer-drivers from the initial 3700 pre-selected mutated genes (Figs 1C and 2). This list consisted of 353 genes already reported in the Catalogue of Somatic Mutations in Cancer (COSMIC), with some already known to be drivers in breast, ovarian and/or prostate cancers (S1 Table–annotation table of the 1537 genes), showing the ability of our pipeline to pick up known drivers.

### Functional properties of candidate genes

**Fig 3:**
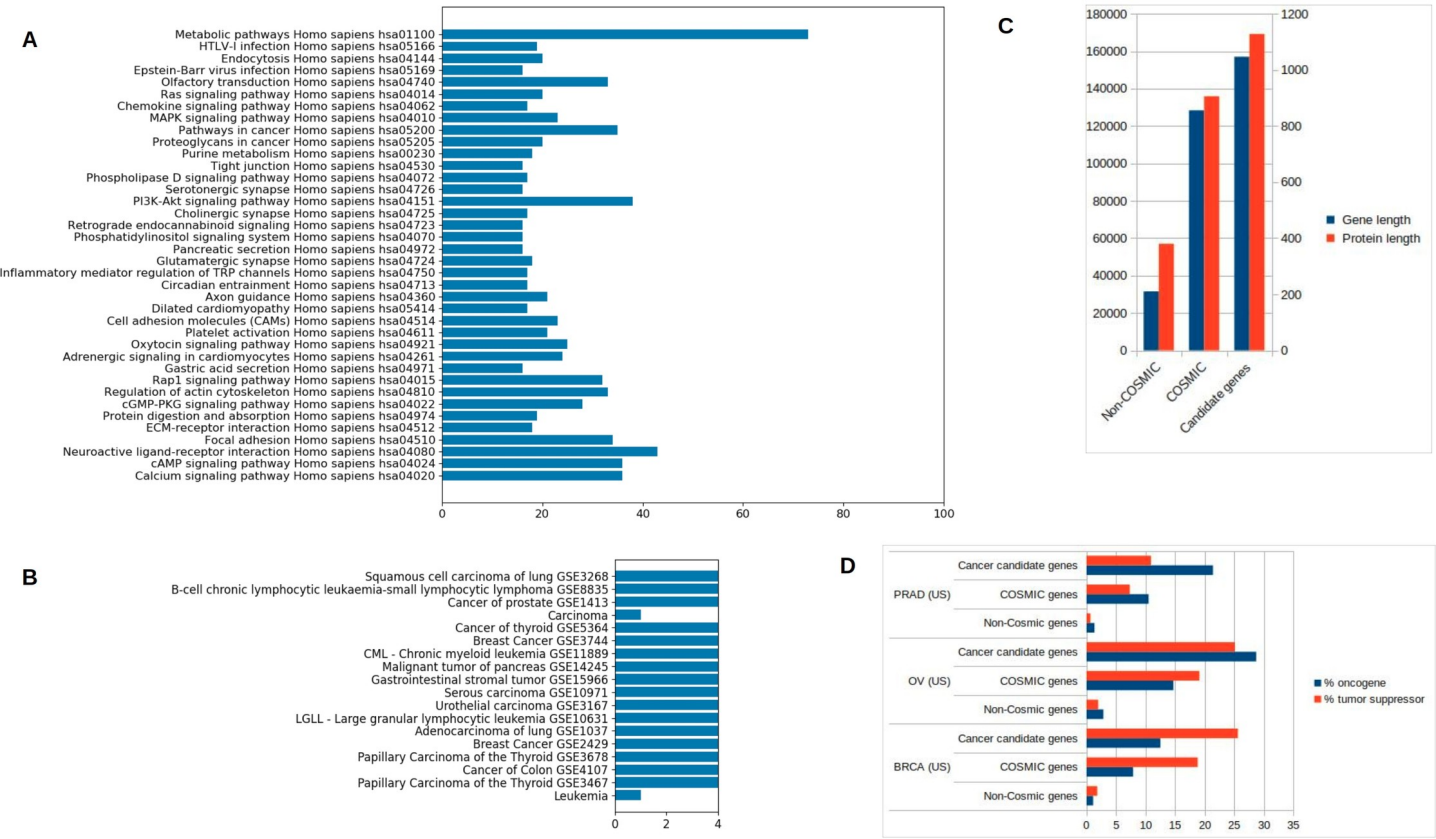
Gene set enrichment & sequences analysis. a) KEGG pathway enrichment for candidate genes, showing the number of genes with specific enrichment for the most enriched pathways; b) Disease signature enrichment showing gene enrichment in cancer-related conditions. c) Gene and protein length comparison between the candidate genes, COSMIC genes and non-COSMIC genes (Gene-length K-S test p-values: candidate vs. non-cancer genes = 0.0, COSMIC vs. non-cancer genes < 0.001; Protein-length p-values: candidate vs. non-cancer genes = 0.0, COSMIC vs. non-cancer genes < 0.001), d) Percentage of oncogenes (blue) and tumor suppressors (red), as defined by the 20/20 rule [3], in the different gene groups within each cancer type (Chi-square tests of results for candidate-genes vs non-COSMIC genes, and COSMIC genes vs non-COSMIC genes: all *p-values* < 0.001 for all cancer types for both oncogene and tumor-suppressor classifications).

To understand the biological effect of our selected candidate cancer-driver genes, gene enrichment analysis was performed on the subset of 1184 non-COSMIC genes in our list of 1537 candidate genes (S1 Table). Gene enrichment analysis matched 555 genes from our list of 1537 candidate genes with KEGG pathway functionalities (S1 Table). Three of those KEGG pathways were directly linked to cancer: pathways in cancer, proteoglycans in cancer, and PI3K-Akt signaling pathway, enriched in approximately 90 of our candidate genes in total (Fig 3A). Disease signature enrichment also revealed that a number of our candidate genes were enriched in cancer-related conditions (S1 Table) (Fig 3B). An analysis of the gene and protein lengths of our candidate genes showed that, on average, these were larger than non-COSMIC genes (presumably, non-cancer genes), and of similar lengths as COSMIC genes, consistent with the knowledge that cancer genes are generally longer [23] (Fig 3C). Finally, when analyzed according to the 20/20 rule defining oncogenes and tumor suppressors [3], both our candidate genes and COSMIC genes had a considerably higher percentage of oncogenes and tumor suppressors than non-cancer genes in all three chosen cancer types (Fig 3D).

### Driver gene discovery in patients with no alterations in COSMIC genes

**Fig 4:**
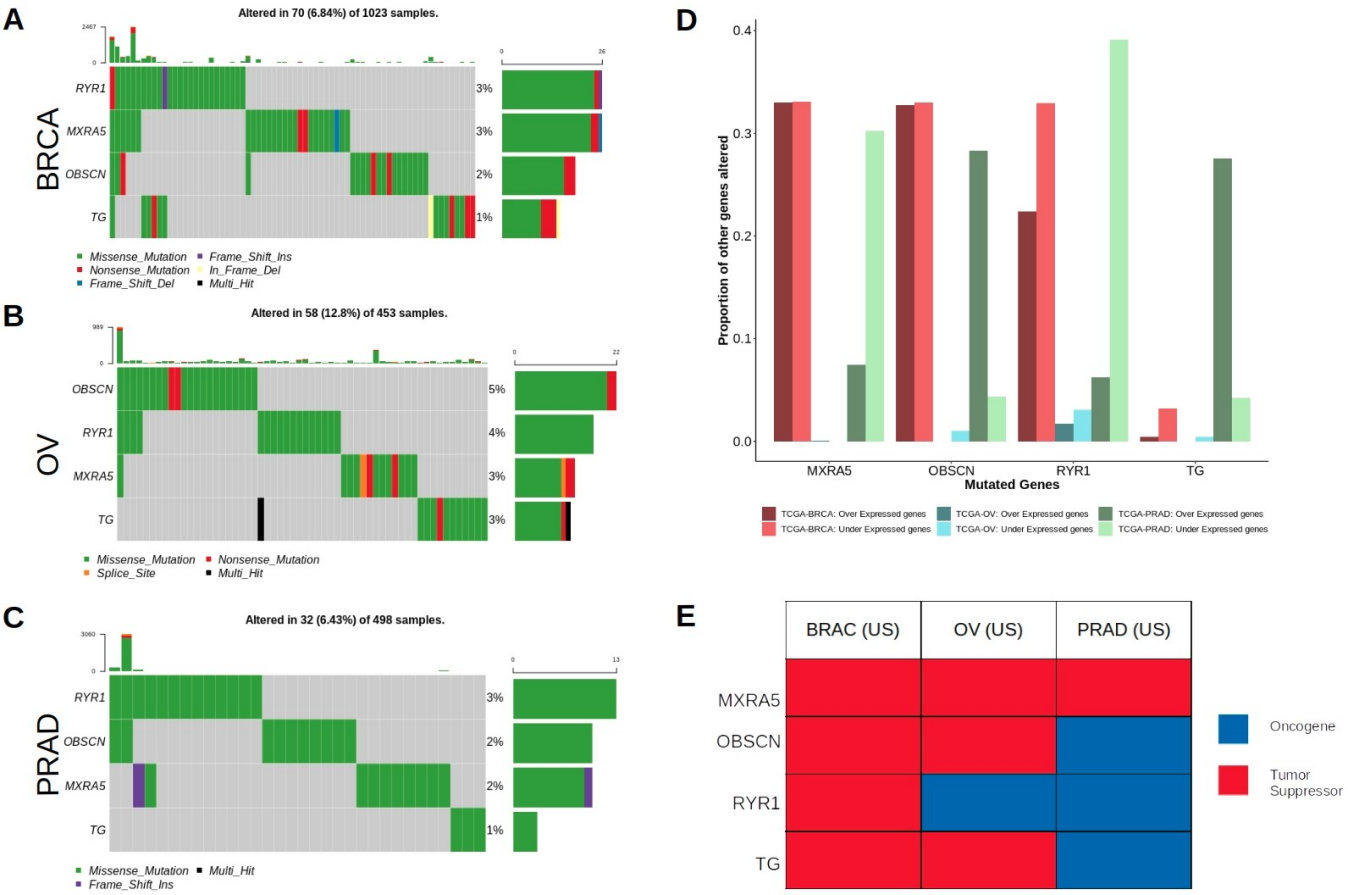
Driver gene discovery in patients with no alterations in COSMIC genes. a-c Oncoplots for 4 significant driver genes discovered in patients with no alterations in COSMIC genes. Oncoplots shown for each gene in our complete datasets (all patients). d) Showing the proportion of genes which experience changes in their expression levels when the four specified genes are mutated in each of the three cancer types–showing both under-expression and over-expression effects. e) Showing the classification as oncogene or tumor suppressor of the four genes in each of our three cancer types.

To ensure that the effects observed above are not merely a result of our candidate genes mutating concurrently with mutations in COSMIC genes, we applied our methodology to a subset of patients harboring no alterations in any COSMIC genes. There were 179 such patients in the BRCA dataset, 163 in the PRAD dataset and 33 in the OV dataset. They had, in common, 67 mutated genes. From this list of 67 pre-selected genes, 4 were found to significantly affect the gene expression of other genes after differential genes expression analysis: *MXRA5*, *OBSCN*, *RYR1* and *TG*. These genes were altered in 6.8%, 12.8%, and 6.4% of patients in BRCA, OV, and PRAD datasets respectively, and harbored mostly missense alterations but also nonsense, frameshift, and splice site mutations (Fig 4A, 4B and 4C). They affected the gene expression patterns of a large number of other genes when mutated (Fig 3D). For example, mutations in *MXRA5* caused > 30% of genes in the BRCA dataset to be over-expressed and again > 30% to be under-expressed, while in PRAD, mutations in this gene caused < 10% of genes to be over-expressed and around 30% to be under-expressed. Mutations in the three other genes had varying, but considerable effects on the gene expression levels of genes in our datasets (Fig 3D). When the 20/20 rule was applied to these 4 genes, results revealed that all of the 4 genes could either be classified as having either tumor suppressor or oncogenic properties in the three cancers (Fig 4E). *MXRA5* had tumor suppressor characteristics in all three datasets, while *RYR1* seems to behave as a tumor suppressor in breast cancer but as an oncogene in prostate and ovarian cancers. *OBSCN* and *TG* seems to both have tumor suppressor properties in breast and ovarian cancers but oncogenic in prostate cancer. Finally, a query of the functional impact of mutations in these 4 genes revealed that they accumulated a number of deleterious and damaging mutations in the patients in our dataset, representing almost half of all the mutations accumulated in those genes (S2 Fig).

## Discussion

The goal of the driver gene discovery method developed here was to use genomic commonalities of cancers occurring at different anatomical sites, intersected with their transcriptomic data, to discover and validate novel cancer driver genes. Using a set of breast, ovarian and prostate cancers, our method produced 1187 putative cancer driver genes, potentially clinically relevant commonly to all three cancer types. Our pipeline identified 553 COSMIC genes at the same time, demonstrating our method is able to also find genes already known to be cancer drivers. In fact, 10 of those COSMIC genes identified, *CDK12*, *CDKN1B*, *CSMD3*, *CTNNB1*, *ERBB2*, *LRP1B*, *MSH2*, *SALL4*, *TP53*, *ZMYM3*, are even to have specific roles in breast, ovarian and prostate cancers (S1 Table).

Almost 90 of the non-COSMIC genes that we identified as potential candidate cancer genes belong to KEGG pathways linked with cancer biology (i.e., pathways in cancer, proteoglycans in cancer, PI3K-Akt signaling pathway), despite not being previously catalogued as COSMIC genes (Fig 3A and S1 Table). Other genes, for which KEGG information is not available, were enriched in various other GO terms, such as ATP binding and apoptosis [24], that linked their functions to tumorigenesis and otherwise cell proliferation or death. Our non-COSMIC candidate cancer genes were also enriched in a number of cancer-related disease signatures (Fig 4B). On average, our candidate genes were larger than non-cancer genes, similar to known COSMIC genes, consistent our expectations of the structure of cancer-causing genes [23] (Fig 4C). A compelling feature of cancer genes is that their oncogenic or tumor suppressor activities can be inferred by the types of variants they accumulate [3]. Following this principle, our list of candidate genes contained more genes with oncogenic or tumor suppressor properties than non-driver genes.

Additionally, our method successfully identified four genes (*MXRA5*, *RYR1*, *OBSCN*, *TG)* that were potential putative driver genes in patients that harbored no mutations in COSMIC genes. This provides high confidence that these four genes were not picked up as a result of co-occurrence with COSMIC genes.

Two of those genes, RYR1 and TG are found in the Candidate Cancer Gene Database (CCGD) [25] after their potential cancer-causing properties were discovered through mouse insertional mutagenesis experiments. The RYR1 has actually been clearly characterized to be downstream of the STAT3 signaling pathway which cause enrichment of breast cancer stem cells, and therefore increases the chances of tumor recurrence or metastasis [26]. Additionally, mutations in TG have been found to be associated with altered sensitivity of a few cancer drugs such as UNC0642, IOX2, and VX-702 [27]. The two other genes, MXRA5 and OBSCN, are enriched in Gene Ontology terms such as ATP-binding and apoptotic signaling, which might affect cell proliferation and therefore tumorigenesis. A recent genomic meta-analysis study found that OBSCN accumulates a number of function-altering mutations in breast cancer samples and reported that OBSCN probably regulates breast cancer tumorigenesis and metastasis through close interactions with other cancer-associated genes involved in breast cancer [28]. The MXRA5 gene, for its part, has been reported as a novel biomarker for colorectal cancer and a predictor of poor prognosis in some types of lung cancer [29, 30]. It has also been found to be significantly upregulated in ovarian cancer, but without a clear indication of its potential role [31].

Our method characterized mutational variation in the genes defined here as candidate cancer drivers as functionally significant following an analysis of their impact on the gene expression profiles of tumor samples. Our method represents a discovery tool that considerably narrows down the search space from tens of thousands of genes to hundreds. It will be important to further test and refine this method with additional data sets, cancer sites, and other validation settings, and to confirm our findings with *in vitro* and *in vivo* models as well as human studies to confirm their causal effect in tumorigenesis and tumor progression.

Most existing methods for driver gene discovery (e.g., MuSiC) rely on identifying recurrent mutations being those that occur at a rate exceeding a background mutation rate [32]. Two main challenges of this one-dimensional approach are 1) the correct estimation of the background mutation rate to minimize false positives [33], and 2) the detection of rare driver mutations. In addition, it has been shown that reliance on mutation frequency to assess the causal status of mutations at a candidate locus may result in genetic misdiagnoses in the germline (and presumably as well in somatic tissue) [21]. Genetic variants can also be characterized as driver mutations if they are within genes that are known to be conserved or that have more signals of positive selection [34]. Yet another way of identifying driver mutations makes use of functionality scores given to mutations based on the type and locations of accumulated variants. Genes having the most cancer-causing effects are shown to exhibit a convergence of functional mutations, called a functional mutation bias [35]. An advantage of this method is its independence from estimated background mutation rates. However, this approach is limited by methods used to score functionality of mutations. Other strategies focus on the frequency of mutations within specific functional regions of the genome, known as hotspot mutations [36]. However, passenger alterations can also occur within hotspot regions [37].

We have referred to the approach proposed here as “agnostic” despite the use of analytical steps that identify putative candidate genes. Using an intersection of mutational and gene expression data ensures that no prior pathway or gene interaction information was needed to generate our candidate genes, which limits bias restricting driver discovery that may be present in other methods. Our method also has the advantage of not relying solely on mutation data (including mutation frequency) for driver gene identification. The differential gene expression analysis results presented indicate that genes with a high mutation frequency do not necessarily correlate with genes having the most significant impact on the expression of other genes. It is well known that rare mutations can be functionally significant. Thus, mutational frequency alone should not be used to infer functionality, which is often the case with methods relying on mutation rate to select cancer drivers. Our method demonstrates the importance of using multi-omics data to distinguish between functional and non-functional genomic variation. A number of other studies have successfully employed the integrative analysis of multi-omics data for the detection of cancer driver genes [38, 39].

Our method is also not without its limitations. We do not consider other factors pertaining to the tumor samples that might be influencing the results. Missing information including race and ethnicity could be an important factor in driver gene selection, given evidence of racial differences in cancer susceptibility that could be attributed to the genomic diversity across populations [40]. It would also be worthwhile to repeat this analysis and taking into consideration tumor grade or stage as well as primary vs. metastatic tumor source when more data become available. Equally, the cohorts analyzed here contained a very limited number of metastatic cases. Stratifying our analysis further to consider these parameters might help to better pinpoint the source of the putative cancer genes identified. Finally, to confirm the relevance of the candidate genes identified here, *in-vitro* methods would need to be performed but are out of scope of this paper, therefore we used *in-silico* methods only such as pathway enrichment, structural analysis and variant properties of the genes, and functionality analyses.

In an era of genome-based precision medicine in oncology, it is crucial to obtain a full picture of driver mutations for predicting prognosis and in therapy development. To date, only 30–40 mutational driver genes had been known for each of our studied cancers, with each tumor containing about 8–10 of these [3]. While some sources argue that the discovery of driver genes has reached a plateau [3], there are still numerous tumors diagnosed with no or too few known mutational drivers [3], highlighting the importance to conduct additional rigorous driver mutation discovery studies using novel methods. We were able to show that with a new intersectional method, there is potential to discover novel cancer-causing candidate genes. The method developed in this study is scalable to other combinations of cancers and genomic data sets. Driver events identified here might have previously been missed when cancer types are considered individually. Such a strategy is particularly pertinent in the repurposing of drugs or the application of a therapies for multiple tumor types based around common mutational events. It is exactly this kind of approach that led to the very recent approval of a new revolutionary class of cancer treatment, Larotrectinib [41]. This drug is said to be tumor-agnostic, meaning it acts against a particular gene mutation (NTRK gene fusion) irrespective of the tumor type (i.e., sarcomas, brain, kidney, thyroid, etc.). Our method has the potential to inform this and other approaches to improve cancer therapeutics and is consistent with current priorities in cancer precision medicine.

## Materials and methods

### Data sources and data preparation

The data for this study were obtained from the publicly accessible The Cancer Genome Atlas (TCGA) [20]. Breast, ovarian, and prostate cancers were selected for this study as a proof of concept because an initial exploration of their mutational profiles revealed that they share about 50% of their top mutated genes in tumor tissue (S1 Fig). We selected TCGA data sets (BRCA-US, OV-US and PRAD-US) for each of the three cancer types. Simple somatic mutation and gene expression (microarray or RNA-seq) data files were downloaded for each data set.

Gene annotations were standardized between all the downloaded data files to the official gene symbol, lists of all mutated genes and tumor samples were extracted, and genes were annotated as being included in the Catalogue of Somatic Mutations in Cancer (COSMIC) or not. The gene expression profiles of each data set were standardized by calculating the z-scores of the gene expression data, in whichever format they were reported (microarray expression values or normalized read counts). Some data sets contained gene expression data reported both from microarray experiments and RNA-seq (S2 Table). Within these two types of expression data, gene expression could be reported as any one of the following: raw read counts, z-scores, or other forms of normalized expression values or read counts (S2 Table). For such data sets, differential gene expression analysis (as explained below) was performed with respect to mutations in the well-established cancer susceptibility genes *BRCA1*, *BRCA2*, and *TP53* as a control experiment to select the most representative files (S2 Table).

### Pre-selection of candidate genes for analysis

To obtain a list of pre-selected candidate genes for consideration in this study, somatic mutation data were processed as follows (Fig 1B). The somatic mutation files, obtained in Simple Somatic Mutation formats, were converted to the Mutation Annotation Format (MAF) using the icgcSimpleMutationToMAF utility of the maftools (R package) [42]. Using the Variant_Classification field of the resulting data, all non-pathogenic mutations were dropped using information from S3 Table, thus leaving only potentially pathogenic mutations. Next, we only considered an intersection set of genes mutated in all three cancer types. This pre-selection of genes was performed on the TCGA data for all three cancer types.

### Integrative data analysis

The consequences of the pre-selected mutated genes were investigated by integrating mutational status and gene expression data. Somatic mutation files for each data set were used to build respective mutation matrices for each data set, denoting the mutational status (mutated/not mutated) of every gene in all samples within the data sets (Fig 1A). Similarly, each gene expression file was used to build an expression matrix for the corresponding data set, denoting gene expression levels of every gene in all samples (Fig 1A).

The significance analysis of microarray (SAM) software, a supervised learning algorithm for genomic expression data mining, was used to perform differential gene expression analysis [43]. A two-class response variable was used to find genes that are differentially expressed with respect to the mutation status of a particular gene across all samples (i.e., for a particular mutated gene, the two classes are defined as mutated or not mutated in the corresponding sample). SAM algorithm measures the strength of the relationship between gene expression and the latter response variable.

The R-based SAM analysis web application (https://github.com/MikeJSeo/SAM) was adapted to a non-web R code for this study. The parameters of the SAM analysis were set to a default false discovery rate (FDR) value (proportion of falsely called genes) of < 0.2. For each pre-selected gene mutated gene, the algorithm computes the statistical comparisons of the mutation status of each sample, with the expression level of all genes across that dataset, and a default threshold is used as cut-off to select differentially expressed genes. The number of genes significantly over-expressed and under-expressed were recorded for all pre-selected genes (Fig 1C). The total number of genes whose expression was affected by each pre-selected mutated gene was compiled and normalized to the total number of genes in the respective datasets.

Following this analysis, the selection of cancer-causing candidate genes was carried out as follows: a gene was selected as a candidate driver gene if it affected the expression of other genes in all three cancer types when mutated (based on SAM analysis results).

### Selection of driver genes not co-occurring with COSMIC genes

In order to select candidate driver genes which have no chance of co-occurrence with COSMIC genes, our method was also applied to a subset of patients (from BRCA-US, OV-US and PRAD-US) who did not harbor any alterations in COSMIC genes.

### Downstream analyses

Following SAM analysis, the genes identified as cancer-associated candidates were subjected to a number of downstream analyses (Fig 1D). First, gene ontology (GO) and pathway enrichment analyses were performed using the ICGC online Data Analysis tool and the Gene Set Enrichment Analysis Python package (gseapy), based on MSigDB (v7.0) [44], to investigate the potential cancer-causing properties of our candidate genes. GO terms for both “Biological Processes” and “Molecular Functions” were considered in our enrichment. For pathway analysis we considered enrichment from the Reactome [45] and the Kyoto Encyclopedia of Genes and Genomes (KEGG) databases [46]. Disease signature enrichment was also performed.

For candidate cancer-drivers, COSMIC genes, and non-cancer genes, an analysis of their average gene and protein lengths were performed as a surrogate for gene conservation. The 20/20 rule, describing an oncogene as a gene having more than 20% missense mutations at the same locus, and a tumor suppressor as a gene having more than 20% truncating mutations [3], was applied to determine the oncogenic and tumor suppressor properties of each of the above groups of genes in each data set. Functional impact of mutations of our final putative driver genes were queried from SIFT and Polyphen-2 calculations in our dataset from the cBioPortal web API [47].

All data processing and analysis, including queries to online bioinformatics databases, were done in Python, R or Bash scripting. Circos plot were generated using the shinyCircos application in R (http://shinycircos.ncpgr.cn/) [48]. Other data visualizations were generated using Python Matplotlib functions or cBioPortal web API [47].

### Statistics

Statistical analysis was done using custom scripts in Python using the python *statistics* packages. Differences between groups were examined either by Kolmogorov–Smirnov test or χ^2^ test. P-values ≤0.001 were interpreted as statistically significant unless stated otherwise.

### Data access

Controlled-access TCGA and ICGC sequence data was approved by NCBI at the US National Institutes of Health (dbGaP Project #20563: “Computational Analysis of Cancer Genomics Data”; Approval Number #76814–1; PI: Shakuntala Baichoo) and by the International Cancer Genome Consortium (ICGC Project #DACO-1067757; “Computational Analysis of Cancer Genomics Data”).

## Supporting information

S1 Fig Distribution of genomics commonalities between the three cancer data sets. Showing the number of distributions of mutated genes common and individual to the data sets.
(DOCX)

S2 Fig Functional impact analysis. Showing the functional impact of mutations in our final putative driver genes (*MXRA5*, *OBSCN*, *RYR1*, *TG*) in each of our datasets, based on Polyphen-2 and SIFT calculations.
(DOCX)

S1 Table Differential gene expression analysis results and annotations. Showing the effect of our selected candidate genes on the over-expression and under-expression of genes in each dataset, reported as the proportion of the total number of genes whose gene expression levels get altered when the specified gene is mutated.
(XLSX)

S2 Table Gene-expression files. Showing results of control experiments (with well-known genes) to select best gene-expression files and normalization of values for further processing. For each data set with multiple gene-expression files, a single best one was chosen to include in expression matrices and downstream differential gene expression. The best file was chosen as the one showing most effect (most numbers of genes affected) on the expression of other genes when three known cancer-drivers are mutated (BRCA1, BRCA2, TP53).
(DOCX)

S3 Table Non-pathogenic mutations. Showing the heuristics used for the elimination of non-pathogenic genes from our dataset.
(XLSX)
