# Supplementary material for: Discovering novel driver mutations from pan-cancer analysis of mutational and gene expression profiles
Source: PLoS One. 2020 Nov 24;15(11):e0242780. doi: 10.1371/journal.pone.0242780 (PMC7685479; doi:10.1371/journal.pone.0242780)
Supplement: S1 Fig — Showing the number of distributions of mutated genes common and individual to the data sets. (DOCX) [file pone.0242780.s001.docx]

### **Genomics commonalities of selected cancer data sets**


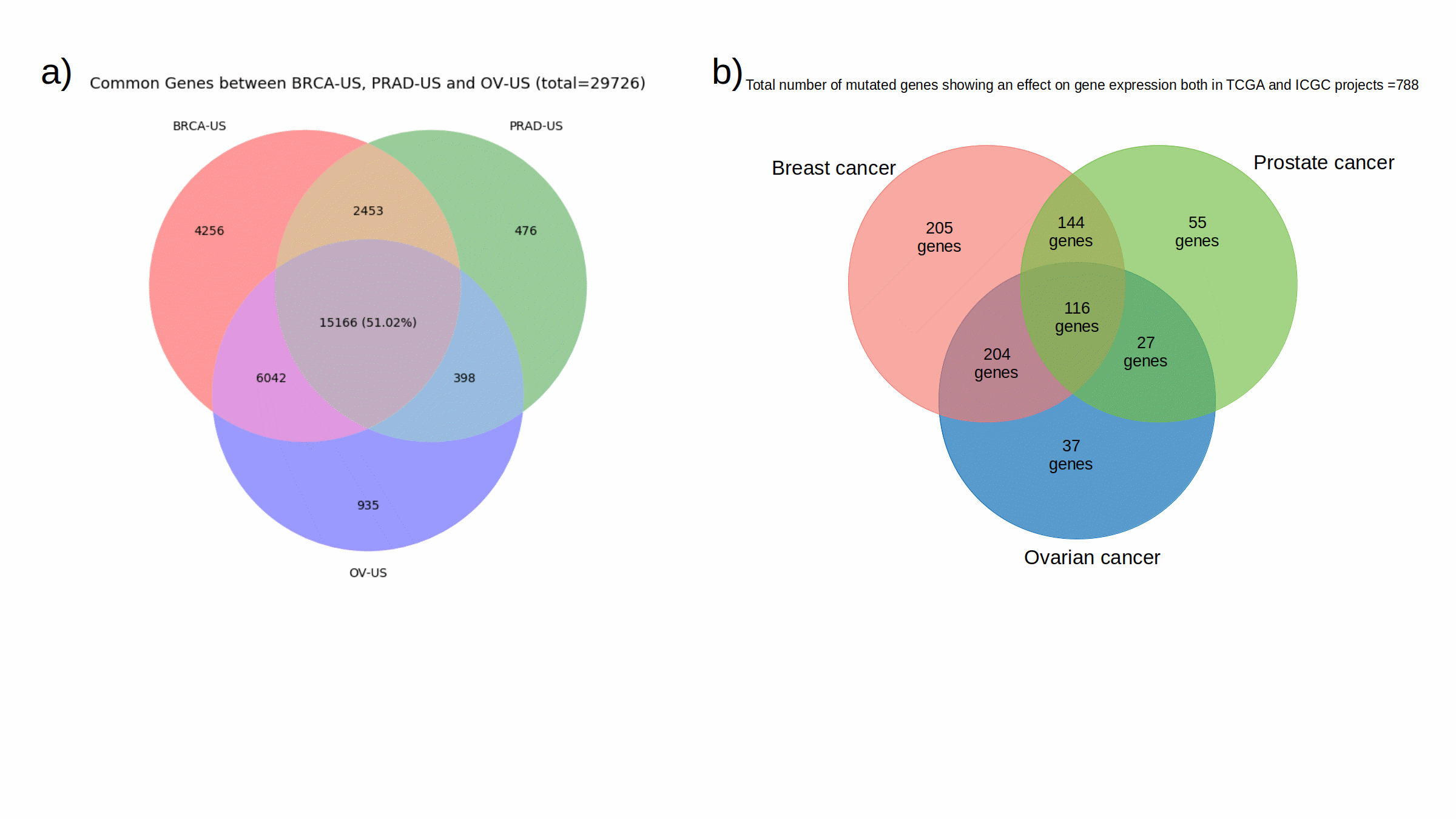


**S1 Fig.** **Distribution of genomics commonalities between the three cancer data sets.** Showing the number of distributions of mutated genes common and individual to the data sets.
