# Supplementary material for: Discovering novel driver mutations from pan-cancer analysis of mutational and gene expression profiles
Source: PLoS One. 2020 Nov 24;15(11):e0242780. doi: 10.1371/journal.pone.0242780 (PMC7685479; doi:10.1371/journal.pone.0242780)
Supplement: S2 Fig — Showing the functional impact of mutations in our final putative driver genes (MXRA5, OBSCN, RYR1, TG) in each of our datasets, based on Polyphen-2 and SIFT calculations. (DOCX) [file pone.0242780.s002.docx]

### **Functional impact analysis of candidate genes**


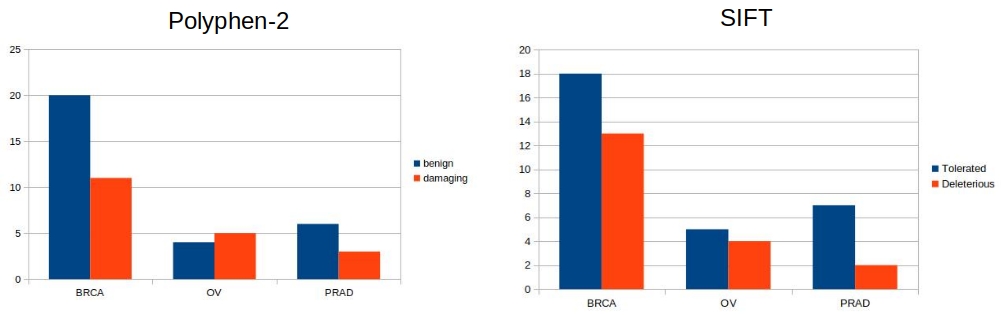
**S2 Fig. Functional impact analysis.** Showing the functional impact of mutations in our final putative driver genes (*MXRA5, OBSCN, RYR1, TG*) in each of our datasets, based on Polyphen-2 and SIFT calculations
