# Supplementary material for: Discovering novel driver mutations from pan-cancer analysis of mutational and gene expression profiles
Source: PLoS One. 2020 Nov 24;15(11):e0242780. doi: 10.1371/journal.pone.0242780 (PMC7685479; doi:10.1371/journal.pone.0242780)
Supplement: S2 Table — Showing results of control experiments (with well-known genes) to select best gene-expression files and normalization of values for further processing. For each data set with multiple gene-expression files, a single best one was chosen to include in expression matrices and downstream differential gene expression. The best file was chosen as the one showing most effect (most numbers of genes affected) on the expression of other genes when three known cancer-drivers are mutated (BRCA1, BRCA2, TP53). (DOCX) [file pone.0242780.s004.docx]

### **Processing of Gene Expression Files**


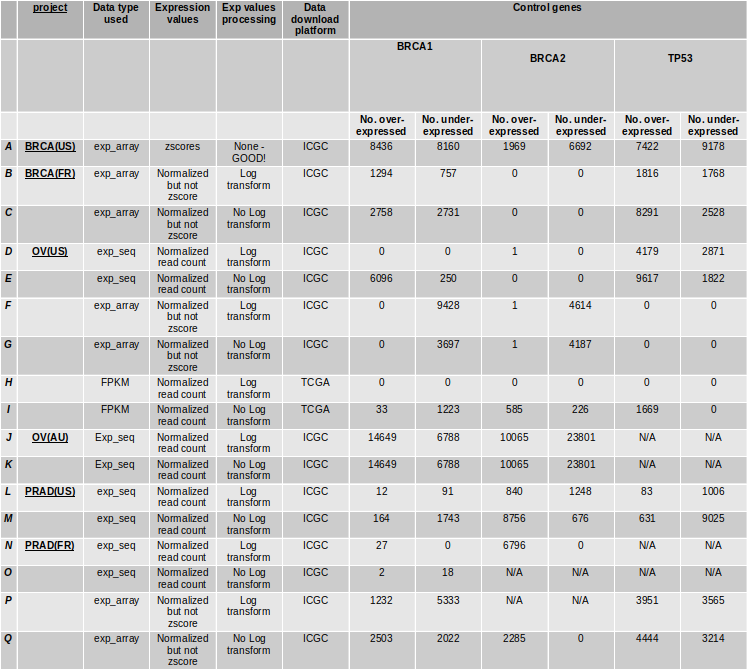


**S2 Table.** **Gene-expression files.** Showing results of control experiments (with well-known genes) to select best gene-expression files and normalization of values for further processing. For each data set with multiple gene-expression files, a single best one was chosen to include in expression matrices and downstream differential gene expression. The best file was chosen as the one showing most effect (most numbers of genes affected) on the expression of other genes when three known cancer-drivers are mutated (BRCA1, BRCA2, TP53).
